# Physicians shortage in primary care: a protocol for updating a systematic review of recruitment and retention strategies

**DOI:** 10.12688/f1000research.174754.1

**Published:** 2026-02-05

**Authors:** Federico Contu, Giulia Cossu, Barbara Bitti, Diego Primavera, Puja Verma, Viviana Forte, John Ford, Mauro Giovanni Carta

**Affiliations:** 1 Department of Medicine Sciences and Public Health, University of Cagliari, Cagliari, Sardinia, Italy; 2 Independent researcher, Cagliari, Italy, Italy; 3 Marylebone Health Centre, London, UK; 4 Wolfson Institute of Population Health, Queen Mary University of London, London, England, UK

**Keywords:** Primary care physicians, Recruitment, Retention, Workforce, High-income countries, Policy, General practice, Family medicine

## Abstract

**Background:**

The global shortage of primary care physicians (PCPs) poses a critical threat to healthcare accessibility and system performance in high-income countries. Previous reviews have documented a wide range of recruitment and retention (R&R) strategies, but the evidence base remains fragmented, outdated, and geographically uneven. In light of demographic transitions, the increasing burden of multimorbidity, and evolving models of care accelerated by the COVID-19 pandemic, an updated synthesis is needed to guide sustainable and context-sensitive workforce policies.

**Methods:**

This systematic review will follow PRISMA (Preferred Reporting Items for Systematic Reviews and Meta-Analyses) 2020 guidelines and is registered with PROSPERO (International Prospective Register of Systematic Reviews) (CRD420251008508). MEDLINE (via PubMed), Embase, and CENTRAL (Cochrane Central Register of Controlled Trials), will be searched for quantitative studies published from February 2015 onward, without language restrictions. Eligible studies will evaluate interventions designed to improve the recruitment and/or retention of PCPs, medical students, or residents in high-income countries. Primary outcomes will include the number of physicians recruited or retained and the duration of retention. Secondary outcomes will cover career intentions, cost-effectiveness, physician satisfaction and well-being, and workforce stability. Two reviewers will independently conduct study selection, data extraction, and risk of bias assessment using validated NIH (National Institutes of Health) quality assessment tools. Findings will be synthesized narratively, with thematic grouping by intervention type, career stage, and contextual factors (e.g., rurality, health system model).

**Discussion:**

This review will provide an updated and comprehensive assessment of R&R strategies for PCPs in high-income countries. By examining intervention logic, outcomes, and contextual modifiers, it will identify which approaches are most effective under specific conditions. The results aim to inform policymakers, educators, and workforce planners in designing targeted, scalable, and context-sensitive strategies to strengthen primary care workforce resilience.

**Systematic Review Registration**: PROSPERO registration number: CRD420251008508.

## Introduction

A global crisis is unfolding as the shortages in the health workforce ^1– 5^ compounded by the unequal distribution ^6– 13^ undermine the foundations of accessible and effective healthcare and create substantial barriers to the equitable delivery of services across populations. ^1, 6, 14, 15^ At the same time, demographic transitions—most notably the population ageing ^16, 17^—and the increasing burden of multimorbidity ^18, 19^ are amplifying pressures on health systems, making the need for strong, resilient, and adequately staffed Primary Health Care more relevant than ever. ^20– 23^

Primary Care Physicians (PCPs) are the cornerstone of healthcare systems, delivering first-contact, comprehensive, continuous and coordinated care. ^24^ A higher density of PCPs correlates with improved population health, increased life expectancy, ^25– 27^ and lower mortality. ^28– 33^ Greater availability of family physicians contributes to earlier cancer diagnoses (breast, ^34^ colon, ^35– 38^ cervical, ^39^ melanoma ^40^) and fewer hospital admissions. ^30, 41^ Continuity of care enhances satisfaction, compliance, and reduces ER visits and hospitalizations. ^42– 46^ Continuity of care and first-contact care also improve efficiency, reducing consultation time, lab test use, and overall healthcare costs. ^47– 50^

The challenges surrounding the PCPs workforce supply–demand imbalance are complex and multidimensional: while shortages have been consistently reported across different countries, ^51– 53^ the unequal distribution of physicians ^6– 13^ amplifies the problem, with particularly severe implications in rural areas and socio-economically disadvantaged urban and suburban settings. ^6, 7, 12, 54– 56^ Already in 1971, Hart formalized this pattern as the inverse care law—‘the availability of good medical care tends to vary inversely with the need for it in the population served’—showing that areas with the greatest morbidity and mortality are served by general practitioners with larger lists, heavier workloads, and less hospital support than healthier areas. ^12^

Several factors have been identified as contributors to the PCP shortages, including increasing workload, ^57, 58^ job dissatisfaction, ^58, 59^ increasing administrative burden ^58, 59^ and a perceived lack of career development opportunities ^58^ leading to leaving practice ^58^ or early retirement. ^59^ Furthermore, multiple studies ^7, 9– 11^ have addressed the determinants of physician’s unequal distribution: a systematic review conducted in 2020 ^13^ explored the factors influencing physician retention in rural and underdeveloped areas, identifying six main categories: financial, professional, working conditions, living conditions, cultural, and personal factors.

In 2010, the WHO issued global policy recommendations to increase access to health workers in remote and rural areas establishing a GRADE-informed framework ^6^ to select, design, implement, monitor, and evaluate rural retention policies, with emphasis on relevance, acceptability, affordability, effectiveness (including complementarities and unintended effects), and impact. Within this context a wide range of recruitment and retention have been documented in multiple reviews, ^54, 55, 60, 61^ including financial incentives, educational interventions, curricular reforms, and policy initiatives to improve career pathways and work environments, with emerging evidence underscoring the importance of multifaceted strategies. ^62^ A recent umbrella review in pre-print in 2025 ^63^ mapped 12 systematic reviews (with searches up to 2019) and found a sparse, geographically concentrated evidence base: the most consistent signals favoured continuous professional development, safe and supportive working environments, and career-development programmes, while effects of financial incentives were mixed and formal evaluations remained limited—thereby underscoring the need for updated, retention-focused syntheses.

The COVID-19 pandemic intensified burnout and attrition and reshaped models of care, generating persistent backlogs and altering the conditions under which PCPs work, ^64^ with rapid shifts in working practices, reduced opportunities for face-to-face care, a widespread use of remote consultations, and a policy-enabled scale-up of telemedicine. ^65^ In parallel Artificial Intelligence is expanding the tools available to clinicians and health systems and may empower new models of care; however, its integration into primary care remains nascent and demands robust governance, workforce training, and equity-focused implementation. ^66, 67^

In this context, a new, comprehensive systematic review is needed to map and appraise current recruitment and retention strategies for PCPs in high-income countries. Our goal is to deliver actionable, evidence-informed insights to policymakers and educators and to guide sustainable, context-aware solutions that bolster primary-care workforce resilience.

### Objectives

This protocol outlines a systematic review of quantitative evaluations of strategies to recruit and retain primary care physicians (PCPs) in high-income countries (HICs), updating the evidence base from 2015 onward to inform policy and training decisions.

The review pursues the following objectives:

1. Determine which strategies—policy, financial, educational/training, regulatory, organizational, and multi-component packages—are associated with improved recruitment and/or retention of PCPs in HICs.
2. Map and compare definitions and measures of “recruitment” and “retention” used across studies and contexts, identifying points of divergence that affect comparability and synthesis.
3. Examine the influence of health-system models (e.g., public vs private/mixed; single- vs multi-payer; gatekeeping arrangements) on the selection, implementation, and effectiveness of recruitment and retention strategies.
4. Assess contextual modifiers—such as rurality/remote status and area-level deprivation—shaping the adoption and effectiveness of recruitment and retention strategies.

## Protocol

### Study design and registry

This review will adhere to PRISMA 2020 ^68^ and has been registered on PROSPERO (CRD420251008508). A populated PRISMA-P checklist is provided as an Extended Data on OSF (DOI 10.17605/OSF.IO/Z6AFJ). We will include studies from high-income countries as listed from the Organization for Economic Co-operation and Development (OECD), and limit inclusion to articles published from February 2015 onward to reflect the contemporary context.

### Review question and PICO

**Table:** 

Component	Description
Population	Primary care physicians (general practitioners, family physicians); Medical students and residents in general practice/family medicine
Intervention	Any intervention explicitly designed to improve recruitment and/or retention in primary care
Comparator	No intervention; Alternative intervention
Outcomes	Primary outcomes: Number/proportion of physicians recruited or retained; Duration of retention Secondary outcomes: Future career intentions; Physician satisfaction and well-being; Workforce stability and accessibility to primary care services; Cost-effectiveness
Study Design	Quantitative studies only

### Eligibility criteria

*Study design*

This systematic review will include any quantitative studies that evaluate the impact of interventions or strategies aimed at improving the recruitment and/or retention of primary care physicians. Given the complexity and diversity of workforce interventions, a broad range of study designs will be considered eligible, including:

• Randomized controlled trials (RCTs) and cluster RCTs
• Quasi-experimental studies, including non-randomized controlled trials
• Controlled before-and-after (CBA) studies
• Interrupted time series (ITS) analyses
• Observational studies, including prospective and retrospective cohort studies, case-control studies, and cross-sectional surveys that report quantitative data on relevant outcomes

Studies must report measurable outcomes related to recruitment or retention. Qualitative studies, mixed-methods studies without extractable quantitative results, opinion pieces, narrative reviews, editorials, and conference abstracts without full text will be excluded.

*Population*

This systematic review will include studies involving individuals at various stages of the primary care medical career pathway. Specifically, eligible populations are:

• Primary care physicians, including general practitioners, family physicians, and other generalist doctors who provide first-contact, continuous, comprehensive, and coordinated care within a primary care setting;
• Medical students enrolled in undergraduate medical programs with a declared or potential interest in primary care;
• Residents or specialty trainees undergoing postgraduate training in general practice or family medicine.

Studies focusing on healthcare professionals other than physicians (e.g., nurses, physician assistants), or on physicians not working in primary care (e.g., specialists), will be excluded.

*Interventions*

This systematic review will include any type of strategy aimed at the recruitment and retention of primary care physicians (General Physicians, General Practitioners, Family Physicians) in high-income countries defined by OECD criteria.

Will be excluded from the review:

• Strategies that do not specifically aim to address the recruitment or retention of primary care physicians or medical students.
• Strategies targeting healthcare professionals other than physicians (e.g., nurses, physician assistants).
• Strategies targeting physicians not working in primary care (e.g., specialists).
• Studies conducted exclusively in low- and middle-income countries, as workforce challenges and healthcare infrastructures in these settings differ significantly from those in high-income countries.
• Studies that do not evaluate a clearly defined intervention or strategy.

*Comparator(s)/Control*

Where relevant, strategies will be compared against alternative strategies or the absence of a specific strategy. Studies with control groups, including comparisons between different interventions or between intervention and non-intervention groups, will be included when available.

*Outcomes and prioritization*

The primary outcomes of interest are:

• The number of primary care doctors recruited or retained, defined as the total number of Primary Care Physicians (including General Practitioners, Family Physicians, or Family Doctors) who enter or remain in primary care practice following a specific intervention.
• The duration of retention, measured in the number of years a physician continues to practice in a given location after a specific intervention.

Additional outcomes will be assessed where reported in the included studies. These may include:

• Future career intentions of physicians, such as the expressed likelihood of entering or remaining in primary care practice.
• Cost-effectiveness, including the reported financial investments associated with recruitment and retention programs, and any available economic evaluations.
• Physician satisfaction and well-being, measured through validated instruments or self-reported assessments.
• Workforce stability and accessibility to primary care services, such as reductions in turnover, increases in workforce supply, or improvements in patient access to general practitioners.

**Table:** 

Outcome measure	Rationale
Number of Primary Care Physicians recruited or retained	Represents the core metric of workforce growth or stabilization following an intervention; directly reflects the primary objective of recruitment and retention strategies.
Duration of retention (years in practice post-intervention)	Measures long-term impact and sustainability of interventions; captures whether strategies lead to meaningful improvements in physician workforce continuity.
Future career intentions	Assesses anticipated workforce behavior, providing predictive insight into long-term retention trends and physician satisfaction.
Cost-effectiveness of strategies	Essential for policy and planning; allows comparison of interventions not only on outcomes but also on resource efficiency and scalability.
Physician satisfaction and well-being	Indicates perceived impact of interventions; correlates with retention and performance; essential to understand intervention impact beyond numerical workforce data.
Workforce stability and accessibility to primary care	Reflects system-level outcomes; helps evaluate whether interventions contribute to equitable service delivery, especially in underserved or rural areas.

*Language*

No language restrictions will be applied. For articles published in languages other than English, translation tools; any issues related to translation will be transparently reported in a specific appendix.

### Information sources

We will search the following electronic databases: MEDLINE (via PubMed), Embase and CENTRAL. The search will cover all literature from February 2015 to the final search date, with no language restrictions. A draft of the search strategy for Embase is reported below. To ensure transparency and reproducibility, the full search strategies will be made publicly available on OSF as Extended Data (DOI 10.17605/OSF.IO/Z6AFJ). ^69^

### Draft search strategy (Embase via Embase.com)

(‘general practitioner’/exp OR ‘general practitioner’ OR ‘family physician’/exp OR ‘family physician’ OR ‘general practice’/exp OR ‘general practice’ OR ‘general practitioner*’:ti OR ‘general practitioner*’:ab OR ‘family physician*’:ti OR ‘family physician*’:ab OR ‘family doctor*’:ti OR ‘family doctor*’:ab OR ‘primary care physician*’:ti OR ‘primary care physician*’:ab OR ‘primary care doctor*’:ti OR ‘primary care doctor*’:ab OR ‘primary care provider*’:ti OR ‘primary care provider*’:ab)

AND

(‘personnel management’/exp OR ‘personnel management’ OR ‘physician engagement’/exp OR ‘physician engagement’ OR ‘retention time’/exp OR ‘retention time’ OR recruitment*:ti OR recruitment*:ab OR retention*:ti OR retention*:ab OR retain*:ti OR retain*:ab OR ‘workforce planning’:ti OR ‘workforce planning’:ab OR ‘physician* allocation’:ti OR ‘physician* allocation’:ab OR ‘workforce retention’:ti OR “workforce retention’:ab OR “workforce stability’:ti OR “workforce stability’:ab OR “physician retention’:ti OR “physician retention’:ab OR “workplace engagement’:ti OR “workplace engagement’:ab) AND [humans]/lim AND [2015-2025]/py

### Search strategy development and peer-review calibration

The strategy will include both controlled vocabulary terms (e.g., MeSH, Emtree) and free-text keywords related to primary care physicians, recruitment, retention, and high-income countries.

The following bibliographic databases will be searched: MEDLINE (via PubMed), Embase (via embase.com), and CENTRAL (Cochrane Central Register of Controlled Trials). The search will cover studies published from 1 February 2015 onwards, with no end-date or language restrictions, to ensure comprehensive coverage of recent evidence. A second reviewer will independently assess the draft strategy for completeness, appropriateness of terms, syntax errors, use of filters, and inclusion/exclusion logic.

Reference lists of all included studies will be hand-searched to identify additional relevant articles not captured through database searching. Duplicate records will be removed in Rayyan. Zotero (v. 7.0.30) will be used as the citation manager for reference handling and manuscript preparation **.** A detailed search log will be maintained to support transparency and reproducibility.

### Study record and selection process

Records retrieved from each database will be exported and imported into Zotero (v. 7.0.30) for reference management. De-duplication will be performed in Rayyan, after which the de-duplicated library will be used for screening and to maintain an audit trail of decisions.

Titles and abstracts will be independently screened by two reviewers (F.C. and B.B.) against the predefined eligibility criteria. Full texts of all studies deemed potentially eligible, or for which there is any uncertainty, will be retrieved and assessed in full.

Any disagreements at the title/abstract or full-text screening stages will be resolved through discussion. If consensus cannot be reached, a third reviewer (D.P.) will be consulted.

The full selection process will be documented using the PRISMA 2020 flow diagram, including the number of records identified, included, and excluded at each phase, with reasons for exclusion clearly reported.

Companion or duplicate publications will be identified through comparison of author lists, sample characteristics, and intervention descriptions, and will be merged or excluded as appropriate to avoid double-counting of data. In cases of missing or unclear data, corresponding authors will be contacted by email (up to three attempts) to request clarification or supplementary information.

### Data extraction

Data will be extracted using a standardised form developed and piloted by the review team. Extracted items will include: study design, country, setting, population, intervention characteristics, comparator (if applicable), outcomes related to recruitment or retention, and key findings. Two reviewers will independently extract data for all included studies; disagreements will be resolved by consensus or, if needed, by a third reviewer (D.P.).

### Risk of bias and quality assessment

Risk of bias will be assessed using:

• The Quality Assessment Tools for Case-Control, Cohort, and Cross-Sectional Studies of the National Institute of Health (USA) will be used.
• The Quality Assessment Tools for controlled Studies of the National Institute of Health (USA) will be used.

Data will be assessed independently by at least two people with a process to resolve differences.

Risk of bias will be assessed at the study level. Risk-of-bias judgements will be used to inform about the certainty of evidence assessment.

### Data synthesis

Given the expected heterogeneity in study designs, interventions, and outcome definitions, findings will primarily be synthesised narratively. We will use a thematic synthesis to categorise the evidence according to the types of recruitment and retention strategies identified, supported by evidence tables and summary matrices. Where helpful, studies will be grouped by: author/year; country; study design; sample characteristics and population/setting (e.g., early-career vs experienced GPs; rural vs urban); healthcare system model (e.g., publicly funded, mixed, private); intervention category (e.g., financial incentives, educational programmes, workforce policies); outcome measure; and results. Quantitative findings will be summarised descriptively (absolute numbers, percentages, and effect estimates as reported). Because substantial variability in methods and outcomes is anticipated, meta-analysis will be considered only when a sufficient number of studies report comparable outcomes and effect estimates can be pooled appropriately; otherwise, results will be presented through narrative synthesis with descriptive statistics.

If quantitative synthesis is feasible, we will conduct a random-effects meta-analysis, selecting effect measures appropriate to the outcome type (RR/OR or MD/SMD). Where needed, we will convert reported statistics to a common effect metric when sufficient information is available, and we will report 95% confidence intervals. Heterogeneity will be assessed using I ^2^; where pooling is not appropriate, findings will be synthesised narratively. Subgroup and sensitivity analyses will be conducted only if feasible, focusing on the prespecified grouping variables (e.g., career stage, setting, intervention category); meta-regression is not planned.

### Meta-bias (es)

Assessment of reporting biases is not planned due to the anticipated heterogeneity and small numbers of studies per outcome. To mitigate potential bias arising from missing or selectively reported results, we will search multiple databases without language restrictions, document the selection process with a PRISMA 2020 flow diagram, extract and report funding sources and authors’ conflicts of interest, and discuss any concerns in the Limitations.

### Confidence in cumulative evidence

The overall certainty (strength) of the body of evidence will be appraised narratively, as prespecified in the PROSPERO record, using criteria aligned with the GRADE domains. For each primary outcome, we will consider: (i) risk of bias of contributing studies (based on the NIH quality assessment tools), (ii) consistency of findings across studies, (iii) precision/imprecision of effect estimates or descriptive summaries, and (iv) directness/applicability to the review question and settings. A formal assessment of publication bias is not planned; any concerns related to missing or selectively reported results will be discussed narratively. Certainty judgements will be summarised in the text and, where helpful, presented in a descriptive summary table (e.g., a narrative ‘Summary of Findings’ table).

### Amendments

Any important amendments to this protocol will be documented in the PROSPERO record, including the date, description, and rationale for each change.

## Conclusions/Discussion

This review will examine recruitment and retention (R&R) strategies for primary care physicians as active components of workforce change, clarifying how they are designed, implemented, and reported—and how they influence career choice, practice location, and tenure in high-income settings. By analysing intervention logic alongside outcomes, and by mapping definitions of “recruitment” and “retention,” the review will identify which elements (e.g., incentives, training pathways, workload redesign, professional support) drive effects, and under what system (financing, gatekeeping) and contextual (rurality, deprivation) conditions they perform best. A structured narrative synthesis will organise findings by strategy type and career stage, translating quantitative results into workforce-relevant metrics to support planning. The resulting evidence will guide the design of more targeted, scalable, and context-sensitive packages, distinguishing core components from adaptable features, and highlighting implementation considerations (acceptability, feasibility, costs) and equity impacts where available. Ultimately, the review aims to inform policy, education, and service planning by providing practical guidance on assembling coherent R&R strategies that strengthen and stabilize the primary-care workforce.

## Dissemination

Results of this review will be disseminated through peer-reviewed publications, conference presentations.

## Ethics and consent

Not applicable. This study does not involve human participants or primary data collection.

## Data Availability

To ensure transparency and reproducibility, the full search strategies will be made publicly available on OSF as Extended Data (DOI 10.17605/OSF.IO/Z6AFJ). ^69^ No data are associated with this article. Open Science Framework (OSF): *Primary care physicians shortage in primary care: An update systematic review of recruitment and retention strategies (protocol).* https://doi.org/10.17605/OSF.IO/Z6AFJ. ^69^ The project contains the following extended data:
• search strategy 12 25.pdf (full database search strategies: MEDLINE/PubMed, Embase, CENTRAL). • Prisma P Checklist.pdf (completed PRISMA-P 2015 checklist). Data are available under the terms of the Creative Commons Attribution 4.0 International license (CC-BY 4.0). **Software:** Reference management and citation handling will be performed using Zotero (v. 7.0.30), and screening (including de-duplication) will be conducted in Rayyan. This protocol follows the PRISMA-P 2015 statement. The completed PRISMA-P checklist is available on OSF as Extended Data (DOI 10.17605/OSF.IO/Z6AFJ; License CC-BY Attribution 4.0 International). ^69^
