# Supplementary material for: Physicians shortage in primary care: a protocol for updating a systematic review of recruitment and retention strategies
Source: F1000Res. 2026 Jun 19;15:194. Originally published 2026 Feb 5. [Version 2] doi: 10.12688/f1000research.174754.2 (PMC13213359; doi:10.12688/f1000research.174754.2)
Supplement: Peer review history

# Reviewer report: Reviewer response for version 2

**DOI:** 10.5256/f1000research.204029.r495285

**Published:** 2026-06-23

**Authors:** Ferdinando Petrazzuoli

**Affiliations:** 1 Lund University, Malmö, Sweden

Thank you to the authors for properly addressing all the points raised in the first release

Is the study design appropriate for the research question?

Yes

Is the rationale for, and objectives of, the study clearly described?

Yes

Are sufficient details of the methods provided to allow replication by others?

Yes

Are the datasets clearly presented in a useable and accessible format?

Not applicable

Reviewer Expertise:

Expert in research in primary care

I confirm that I have read this submission and believe that I have an appropriate level of expertise to confirm that it is of an acceptable scientific standard.

# Reviewer report: Reviewer response for version 1

**DOI:** 10.5256/f1000research.192678.r486296

**Published:** 2026-05-26

**Authors:** Ferdinando Petrazzuoli

**Affiliations:** 1 Lund University, Malmö, Sweden

**1. Constructive assessment for the authors**

 This manuscript presents a protocol for an updated systematic review of recruitment and retention strategies for primary care physicians in high-income countries. The topic is timely and important, given persistent primary care workforce shortages, unequal geographical distribution of physicians, increasing demand from ageing and multimorbidity, and changes in care delivery following COVID-19.

 The rationale is clear and well supported. The authors effectively explain why an updated synthesis is needed, particularly because previous reviews are now dated, geographically uneven, and may not fully reflect recent workforce pressures, digital transformation, or post-pandemic working conditions. The objectives are relevant and appropriately aligned with the proposed review question.

 The protocol is generally well designed. It is registered in PROSPERO, follows PRISMA guidance, uses broad but justified eligibility criteria, and includes independent screening, extraction, and risk-of-bias assessment by two reviewers. The planned narrative synthesis is appropriate given the expected heterogeneity in interventions, outcomes, and study designs. The inclusion of contextual modifiers such as rurality, deprivation, and health-system model is a strength, as recruitment and retention interventions are highly context dependent.

 I have several recommendations that could further strengthen the protocol:

 First, the authors could provide clearer operational definitions of “recruitment” and “retention.” Although the protocol states that definitions will be mapped across studies, pre-specifying categories or minimum criteria would improve consistency during screening, extraction, and synthesis.

 Second, the authors should clarify the criteria for deciding whether meta-analysis is feasible. The protocol currently states that meta-analysis will be considered where outcomes are sufficiently comparable, but more detail would be useful, for example regarding minimum numbers of studies, acceptable outcome similarity, and how heterogeneity will influence decisions.

 Third, the authors may wish to include a simple conceptual framework linking intervention categories, mechanisms of action, intermediate outcomes, and final workforce outcomes. This would help readers understand how financial, educational, regulatory, organisational, and multi-component strategies are expected to influence recruitment and retention.

 Fourth, the planned assessment of certainty of evidence could be described more explicitly. The protocol mentions GRADE-aligned domains, but a more structured approach, even if narrative rather than fully formal GRADE, would improve transparency.

 Finally, given the broad scope of the review, the authors could explain how they will prioritise findings if the evidence base is too heterogeneous or extensive. For example, they could pre-specify that primary outcomes, physician-level retention/recruitment outcomes, or higher-quality comparative studies will receive greater interpretive weight.

 I was not able to assess the underlying results or conclusions of the planned systematic review, as this is a protocol and no review findings are yet available. I also could not assess the performance of the final search strategy across all databases beyond the information provided in the manuscript and linked supplementary materials.

**2. Mandatory reviewer questions**

**Is the rationale for, and objectives of, the study clearly described?**

 Yes. The rationale is well developed and the objectives are clearly stated, relevant, and aligned with the research question.

**Is the study design appropriate for the research question?**

 Yes. A systematic review is appropriate, and the inclusion of diverse quantitative study designs is justified by the nature of workforce interventions.

**Are sufficient details of the methods provided to allow replication by others?**

 Yes, with minor reservations. The eligibility criteria, databases, screening process, data extraction, risk-of-bias assessment, and synthesis approach are described in sufficient detail. Replicability would be improved by more detail on meta-analysis feasibility criteria, operational outcome definitions, and the structured application of certainty-of-evidence assessment.

**Are the datasets clearly presented in a usable and accessible format?**

 Not applicable. This is a study protocol and no dataset has yet been generated.

**3. Approval status**

**Approved with reservations.**

 The protocol is scientifically sound and suitable for indexing, but the minor methodological clarifications above would improve transparency, reproducibility, and usefulness of the eventual review.

**4. Suggested statement to invite the authors to include**

 I invite the authors to include the following statement in the manuscript:

 The authors should clarify that any amendments to the protocol, including changes to eligibility criteria, outcomes, search strategy, or synthesis methods, will be documented transparently in PROSPERO and reported in the final review, with dates and rationales for each amendment.

Is the study design appropriate for the research question?

Yes

Is the rationale for, and objectives of, the study clearly described?

Yes

Are sufficient details of the methods provided to allow replication by others?

Yes

Are the datasets clearly presented in a useable and accessible format?

Not applicable

Reviewer Expertise:

Expert in research in primary care

I confirm that I have read this submission and believe that I have an appropriate level of expertise to confirm that it is of an acceptable scientific standard.

# Response

**Published:** 2026-06-15

**Authors:** Federico Contu

**Dear Dr Petrazzuoli,**

 Thank you for your careful and constructive appraisal of the protocol, and for your positive overall assessment. We have considered each of your recommendations, and respond point by point below.

**Criteria for meta-analysis feasibility.**

 We agree and have addressed this point. We have expanded the Data synthesis section to specify that meta-analysis will be undertaken only where at least two studies report comparable outcomes amenable to pooling, using a random-effects model. We have further clarified how heterogeneity, assessed via the I² statistic and interpreted together with judgements on the clinical and methodological comparability of studies, will inform the decision between quantitative pooling and narrative synthesis.

**Documentation of protocol amendments.**

 We have adopted this recommendation. We have aligned the Amendments section of the protocol with your suggested wording, clarifying that any important amendments — including changes to eligibility criteria, outcomes, search strategy, or synthesis methods — will be documented transparently in the PROSPERO record and reported in the final review, with the date and rationale for each change.

**Operational definitions of "recruitment" and "retention".**

 As one of the primary objectives of the review (Objective 2) is to map and compare the definitions and measures of "recruitment" and "retention" across studies and contexts, pre-specifying operational definitions at the screening stage would risk constraining the diversity of definitions the review aims to capture, and could lead to the exclusion of relevant studies adopting alternative conceptualisations. We have therefore retained our planned mapping approach, and we will build on its findings to propose operational definitions and a categorisation framework within the final systematic review.

**Conceptual framework, structured certainty-of-evidence assessment, and prioritisation of findings.** As these refinements relate to the synthesis and interpretation of results rather than to the scientific soundness of the protocol, we will incorporate them at the appropriate stage of the final systematic review — including a conceptual model linking intervention categories, mechanisms of action, and workforce outcomes; a more structured, GRADE-informed narrative assessment of certainty for each primary outcome; and pre-specified criteria for prioritising findings (for example, greater interpretive weight to primary outcomes, physician-level recruitment and retention outcomes, and higher-quality comparative studies) should the evidence base prove broad or heterogeneous. We will keep these points in mind throughout the conduct of the review.

 We thank you again for your comments, which have strengthened the protocol.

# Reviewer report: Reviewer response for version 1

**DOI:** 10.5256/f1000research.192678.r466849

**Published:** 2026-04-23

**Authors:** Ana Isabel González González

**Affiliations:** 1 Consejería de Sanidad, Comunidad de Madrid, Madrid, Spain

**SUMMARY**

 This manuscript presents a protocol for a systematic review aiming to update the evidence on recruitment and retention (R&R) strategies for primary care physicians (PCPs) in high-income countries.

 The authors justify the need for this work based on persistent and worsening global shortages of PCPs, increasing demand due to population ageing and multimorbidity, limitations of existing evidence (fragmented, outdated, geographically uneven) and system-level changes (COVID-19, digital health, AI)

 The planned review will follow PRISMA 2020 guidelines and has been registered in PROSPERO. It will include quantitative studies (2015 onward) from MEDLINE, Embase, and CENTRAL and will evaluate interventions targeting recruitment and/or retention. The review will focus on outcomes such as: Number of physicians recruited/retained, duration of retention, career intentions, satisfaction, cost-effectiveness, and system-level outcomes

 The methodology includes: Dual independent screening and extraction, risk of bias assessment using NIH tools, primarily narrative synthesis, with meta-analysis if feasible and consideration of contextual factors (e.g., rurality, health system models)

 The study aims to produce policy-relevant, actionable insights for workforce planning.

**COMMENTS**

**Rationale and objectives**

 Assessment: YES

 The rationale is clear, well-structured, and compelling. It integrates epidemiological, health system, and policy arguments. It explicitly identifies gaps in previous reviews (e.g., outdated evidence, lack of contextualisation).

 The objectives are clearly stated, structured into four distinct and relevant aims, directly aligned with the research problem

*Minor suggestion* (not required for scientific soundness): The authors could prioritise objectives (primary vs secondary aims) to improve clarity and focus.

**Study design**

 Assessment: YES

 The choice of a systematic review is fully appropriate for the research question.

 The design is methodologically sound because it follows established standards (PRISMA, PROSPERO), it includes a broad range of quantitative designs, which is justified given the complexity of workforce interventions and the expected lack of RCTs in this field.

*Minor suggestions*

 Clarify decision rules for meta-analysis feasibility (currently somewhat vague)

 Consider explicitly stating how heterogeneity thresholds (e.g., I²) will influence decisions

**Methods and reproducibility**

 Assessment: YES

 The methods are described in sufficient detail to allow replication.

 Key elements supporting reproducibility: Clear eligibility criteria (Population, Intervention, Outcomes, Study design), explicit inclusion/exclusion criteria, detailed search strategy (example provided), transparent screening and selection process (dual reviewers, conflict resolution), defined data extraction variables, risk of bias tools specified, planned synthesis methods described.

 The inclusion of OSF repository and PRISMA-P checklist further strengthens transparency.

*Minor suggestions*

 Provide more detail on: Data extraction form (template or variables list in supplement), handling of missing data and unclear reporting and clarify whether calibration exercises will be conducted among reviewers

 These are improvements, not requirements for scientific validity.

**Other minor comments and suggestions**

 These do not affect scientific soundness, but could improve clarity and impact.

 The manuscript would benefit from improved conceptual clarity through the inclusion of a clearer framework explicitly linking intervention types, their underlying mechanisms of action, and the expected outcomes. While these elements are all present in the protocol, they are not fully integrated into a single conceptual model. Making these relationships more explicit would strengthen the interpretability of the future synthesis and help readers better understand how different strategies are expected to influence recruitment and retention outcomes across contexts.

 Regarding outcome definitions, although the manuscript clearly lists primary and secondary outcomes, further standardisation would enhance consistency. In particular, key concepts such as “recruitment” and “retention” may vary substantially across studies in terms of definition and measurement. The authors already acknowledge this issue and plan to map definitions across studies, which is a notable strength. However, the protocol could be further improved by pre-specifying operational definitions or categorisation frameworks in advance, thereby facilitating more structured comparisons and synthesis.

 The scope of the review is appropriately ambitious but may raise feasibility challenges. The inclusion of multiple study designs, a wide range of intervention types, and numerous outcomes is justified given the complexity of workforce interventions; however, this breadth is likely to result in substantial heterogeneity. To address this, the authors should clarify how prioritisation will be managed if the evidence base proves too broad or heterogeneous to synthesise meaningfully. For example, outlining criteria for focusing on specific intervention categories, outcomes, or study designs would strengthen the methodological clarity.

 In terms of bias and certainty of evidence, the planned use of NIH quality assessment tools is appropriate and aligned with the inclusion of diverse study designs. However, the approach to assessing the overall certainty of evidence using GRADE is described only narratively. The protocol would benefit from a more structured application of GRADE, even if simplified, to ensure transparency and consistency in evaluating the strength of the evidence across outcomes.

 No issues have been identified that must be addressed to ensure the scientific soundness of the study. Overall, the protocol is methodologically robust and suitable for indexing in its current form.

 In conclusion, this is a scientifically sound protocol of high methodological quality. It addresses an important and timely topic, demonstrates strong methodological rigour, and adheres to best practices in transparency and reproducibility. The manuscript is well-designed and clearly reported, and the proposed review has the potential to make a meaningful contribution to the field.

 Final verdict: the manuscript is acceptable as it stands, with only minor improvements suggested.

Is the study design appropriate for the research question?

Yes

Is the rationale for, and objectives of, the study clearly described?

Yes

Are sufficient details of the methods provided to allow replication by others?

Yes

Are the datasets clearly presented in a useable and accessible format?

Not applicable

Reviewer Expertise:

Healthcare services research. Multimorbidity.

I confirm that I have read this submission and believe that I have an appropriate level of expertise to confirm that it is of an acceptable scientific standard.

# Response

**Published:** 2026-06-15

**Authors:** Federico Contu

*Dear Dr González González,*

 Thank you for your careful review, and for considering the protocol scientifically sound and suitable for indexing in its current form. We have considered each of your suggestions and respond point by point below.

**Meta-analysis feasibility and heterogeneity thresholds.**

 We agree with this point and have addressed it. We have expanded the Data synthesis section to specify the decision rules for quantitative synthesis: meta-analysis will be considered only where at least two studies report comparable outcomes with effect estimates that can be appropriately pooled, using a random-effects model. We have also clarified that statistical heterogeneity will be assessed using the I² statistic, interpreted alongside clinical and methodological judgement on comparability, and that where heterogeneity is substantial or pooling is otherwise inappropriate, findings will be synthesized narratively.

**Operational definitions of "recruitment" and "retention".**

 We would respectfully note that one of the primary objectives of this review (Objective 2) is precisely to map and compare the definitions and measures of "recruitment" and "retention" used across studies and contexts, and to identify the points of divergence that affect comparability. Pre-specifying operational definitions at the screening stage would risk constraining the very heterogeneity of definitions that the review seeks to capture, potentially excluding relevant studies that conceptualize these outcomes differently. For this reason, we have retained our planned approach of mapping definitions across the included studies. We recognize the value of standardization, and we will draw on the resulting mapping to propose operational definitions and a categorization framework as part of the final systematic review.

**Conceptual framework, structured GRADE assessment, data extraction form, handling of missing data, and reviewer calibration.**

 As these refinements pertain primarily to the synthesis and reporting of results rather than to the scientific validity of the protocol itself, we will incorporate them at the appropriate stage of the final systematic review — including a conceptual model linking intervention types, mechanisms, and outcomes; a more structured, GRADE-informed narrative appraisal of certainty per outcome; and fuller documentation of the data extraction form, the handling of missing or unclear data, and the inter-reviewer calibration exercise. We will keep these points in mind throughout the conduct of the review.

 We thank you again for your feedback, which has strengthened the protocol.
